# Outcomes of the Ponseti Technique in Different Types of Clubfoot—A Single Center Retrospective Analysis

**DOI:** 10.3390/children10081340

**Published:** 2023-08-03

**Authors:** Maryum Naseer Butt, Wajida Perveen, Carmen-Iulia Ciongradi, Dan Iulian Alexe, Misbah Marryam, Laique Khalid, Daniel Lucian Dobreci, Ioan Sârbu

**Affiliations:** 1 Muzaffarabad Physical Rehabilitation Centre, Muzaffarabad 13100, Pakistan; mbutt_ajk@yahoo.com; 2 School of Allied Health Sciences, CMH Lahore Medical College & IOD (NUMS Rawalpindi), Lahore 54810, Pakistan; 3 2nd Department of Surgery—Pediatric Surgery and Orthopedics, “Grigore T. Popa” University of Medicine and Pharmacy, 700115 Iasi, Romania; sarbu.ioan@umfiasi.ro; 4 Department of Physical and Occupational Therapy, “Vasile Alecsandri” University of Bacau, 600115 Bacau, Romania; alexedaniulian@ub.ro (D.I.A.); dobreci.lucian@ub.ro (D.L.D.); 5 Benazir Bhutto Hospital, Rawalpindi 23000, Pakistan; physiomisbah@gmail.com; 6 Combined Military Hospital, Muzaffarabad 13100, Pakistan; laiquekhalid1989@gmail.com

**Keywords:** clubfoot, talipes equinovarus, Ponseti, tenotomy

## Abstract

**Background**: Clubfoot is a congenital deformity that can affect one or both of a newborn’s lower extremities. The main objective of the study is to evaluate and compare the outcomes of the Ponseti method for the management of different types of clubfoot. **Methods**: A retrospective analysis of 151 children with 253 clubfeet (idiopathic untreated, idiopathic recurrent, and syndromic) with at least one year of follow-up was conducted in four months after ethical approval. Data were collected with a structured proforma after the consent of the parents. An independent sample *t*-test was applied to show the comparison between the groups, and a *p*-value of 0.05 was considered significant. **Results**: Out of 151 patients, 76% were male and 24% were female. Out of a total of 235 feet, 96 (63%) were idiopathic untreated, 40 (26.5%) were idiopathic recurrent, and 15 (9.5%) were syndromic clubfoot. The average number of casts was higher in syndromic clubfoot (9 casts per foot). There was no significant difference in the baseline Pirani score of the three groups (*p*-value > 0.05); but after one year of follow-up, there was a significant difference in the Pirani score of idiopathic and syndromic clubfoot (*p*-value ≤ 0.05) and between recurrent clubfoot and syndromic clubfoot (*p*-value = 0.01). **Conclusions**: The aetiology of syndromic clubfoot affects the outcomes of the Ponseti method and leads to relapse. In idiopathic (untreated and recurrent) clubfoot, the Ponseti method does not produce a significant difference in outcome. Poor brace compliance and a lack of tenotomy lead to orthotic (ankle foot orthosis AFO and foot orthosis FO) use in the day time and the recurrence of clubfoot deformity in these three types of clubfoot.

## 1. Introduction

Clubfoot, or congenital talipes equinovarus (CTEV), is the most common musculoskeletal birth anomaly, with an estimated 6000–7000 cases per year in Pakistan [1]. The incidence of clubfoot fluctuates around the world. It has an incidence of 1.5 per 1000 live births in Pakistan [1], 0.9/1000 live births in India [2], 1/1000 live births in the USA [2], 0.87/1000 live births in Japan [2], and 3.49/1000 live births in Australia [2]. The male-to-female ratio is 2.5 to 2.8:1 [1,3]. The word talipes equinovarus derives from ‘tali’, which means ankle, ‘pes’, which means foot, ‘equinus’, which means pointed downward or horse foot, and ‘varus’, which means towards the midline [1]. The cause of clubfoot is not very well understood to date. There are many factors like environmental influence, genetic variations, and the position of the foetus in utero that play a part in the development of clubfoot deformity. Several theories have been proposed, but they have not been proven to date [3]. A mechanical theory has been proposed suggesting that a reduction in amniotic fluid can make the environment inside the uterus sensitive to external forces, which can lead to foot deformities. In contrast, the theory of neuromuscular defects proposes that there is a neuromuscular balance in the extrinsic muscles of the foot, which leads to idiopathic congenital clubfoot in the foetus [4].

Clubfoot is classified into primary (idiopathic) and secondary clubfoot. The idiopathic clubfoot is further divided into neglected, recurrent, resistant, complex, and atypical clubfoot. Idiopathic untreated clubfoot is a type that has not received any treatment until the time of the first presentation at the hospital. Idiopathic treated clubfoot is a type that is successfully treated with the Ponseti method until the age of 5 years. Recurrent clubfoot is a type in which the foot deformity re-occurs after successful Ponseti treatment. Neglected clubfoot is a type of clubfoot that has not been treated until the age of walking, usually until the age of 2 years. Complex clubfoot is a type in which procedures other than the Ponseti method have been performed that make the foot deformity more complex than the initial deformity. Resistant clubfoot is a type in which the deformity does not respond to the Ponseti method. Atypical clubfoot is defined as a foot deformity with a chubby, swollen foot, a planter-flexed first metatarsal ray, and an extended big toe [3,5].

Secondary clubfoot is non-idiopathic and usually occurs secondary to other neurological or musculoskeletal disorders [3,6,7]. It usually has associated spina bifida, tethered cord syndrome, and arthrogriposis multiplex congenita [3,7,8,9].

The diagnosis is usually performed prenatally in the ultrasound scan at 20–22 weeks, [10,11]. If it is not detected in the scan, the diagnosis is purely based on the clinical presentation after the birth of the child. The deformity of clubfoot is further divided into four individual deformities known as ‘’CAVE’’ deformities [3,12]: C for cavus, which means high arched; A for metatarsal adductus; V for hindfoot varus; and E for equinus [3]. The presence of these four deformities confirms the clubfoot. The severity of clubfoot deformity is measured by using the Pirani score, which is a simple and reliable tool. This scoring system allows the clinicians to assess not only the severity of the deformity but the outcomes of treatment as well [3,13,14]. The Pirani score assesses the hindfoot and midfoot components of deformity. The hindfoot score consists of three components: posterior crease, rigid equinus, and empty heel. The midfoot score consists of three components: the lateral head of the talus, the medial crease, and the curving lateral border [3]. Each component is scored according to the three-point scale. If the sign is severe, then it scores as 1; if the sign is partial, then it scores as 0.5; and if it is normal, then it scores as 0. Its total score ranges between 0 and 6 for midfoot and hindfoot both. The lower score means the deformity is less severe and vice versa. The examiner takes a seated position with the child in the parent’s lap. The procedure should be painless and performed with the least effort [3,13,14].

There have been many treatment strategies used for the management of clubfoot, but Ponseti developed and defined a method that is considered to be the gold standard. The Ponseti method consists of two phases: the corrective phase, which includes serial casting followed by percutaneous tenotomy, and the maintenance phase, which includes a foot abduction brace. It is a cost-effective treatment and aims to produce functional, pain-free, and normal-looking feet. After successful treatment, a child can wear normal shoes and walk without a permanent disability [5]. Pavone V et al. reported that children treated with the Ponseti method become able to walk independently within the normal age limit for the milestone but later than the healthy controls [15].

Shah et al. found that the Ponseti method is the best way to manage the clubfoot and only very rare cases require surgical intervention [16].

The main purpose of this study is to evaluate and compare the outcomes of the Ponseti method for the management of three different types of clubfoot (idiopathic, recurrent, and syndromic). Furthermore, this study will also focus on the evaluation of factors that affect the outcome of the Ponseti method.

## 2. Materials and Methods

We performed a comparative retrospective analysis at the Muzaffarabad Physical Rehabilitation Center after ethical approval (No. 973/Admin 2018 dated 25 May 2018). As this was a retrospective study, we have chosen a non-probability-convenient sample of 151 children with clubfoot. The sample was based on a review of patient files. Data were taken solely from those files which were complete. No formal calculation for sample size could be applied.

### Inclusion and Exclusion Criteria

Children with idiopathic, recurrent, or syndromic clubfoot with at least 1 year of follow-up were included in the study. Children with neglected, atypical, and complex feet, or with co-morbid conditions like DDH and cerebral palsy were excluded.

**Figure 1:**
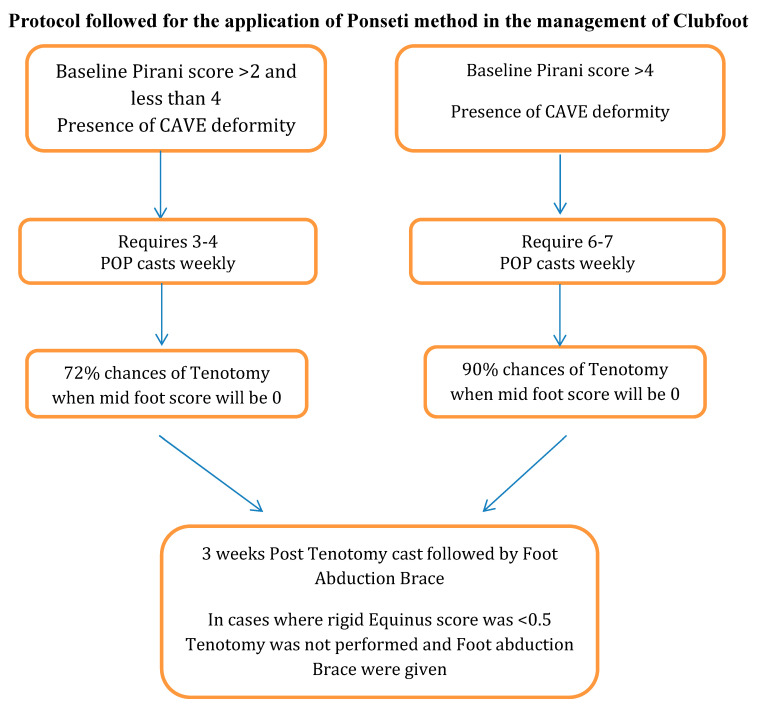
Demonstrating the protocol followed for the application of the Ponseti method.

All the participants were treated at the same centre, casting was applied by a physiotherapist and children were referred to the same Ponseti certified orthopaedic surgeon outside the study centre. Data were collected using a self-structured questionnaire from the records of the centre, with the consent of the parents or guardians, by a physiotherapist who is a certified Ponseti practitioner. The severity of the CTEV was assessed through the Pirani score. The protocol followed for treatment of the cases is given in Figure 1. The children who presented with a clubfoot deformity and had an initial Pirani score of more than 2 but less than or equal to 4 had a 72% higher chance of receiving a tenotomy in the future. But children who presented with a clubfoot deformity and had an initial Pirani score of more than 4 had a 90% chance of receiving a tenotomy in the future. Finally, in cases where the Pirani score for rigid equinus was less than 0.5, a tenotomy was not performed.

The data were analysed using SPSS-21. For the 151 samples, the percentage of each type of clubfoot was calculated. A one-way analysis of variance was conducted to determine the significant difference between the three groups. An independent sample *t*-test was applied to show the comparison between the groups, and a *p*-value of 0.05 was considered significant.

## 3. Results

In total, 151 children with 235 club feet were treated with the Ponseti method, and a two-year follow-up was done. The ratio of idiopathic clubfoot was 63%, recurrent clubfoot was 26.5%, and syndromic clubfoot was 9.5%. Males made up 76% of the population, while females made up 24%. The main difference seen in the three types of clubfoot was the average number of casts per foot, which was nine, four, and five for syndromic, idiopathic, and recurrent cases, respectively. The baseline characteristics of the participants are given in Table 1.

There was no significant difference in the baseline Pirani score of the idiopathic untreated clubfoot, idiopathic recurrent clubfoot, and syndromic clubfoot groups (*p*-value > 0.05). After one year of follow-up, there was a significant difference in the Pirani score among all three groups (*p*-value ≤ 0.05), and there was a significant difference within all three groups as well (*p*-value = 0.01) (Table 2).

A total of 90 (59.5%) children with clubfoot experienced relapses in deformity during different phases of treatment. In the corrective phase, 28 (18.5%) children with clubfoot experienced wounds with broken casts and skin allergies because of poor hygiene of the cast at home, due to which cast holidays were given and children with clubfoot experienced relapses with clubfoot. In the maintenance phase, 62 (41%) children with clubfoot experienced relapse due to poor brace compliance.

Of 151 children, 39 (25.8%) children were treated with tenotomy, a *t*-test was applied to measure the differences of the Pirani score in children treated with and without tenotomy at baseline, after serial casting or before the tenotomy, at the time of application of a foot abduction brace, and after 1 year of follow-up since the first visit (Table 3). There was a significant difference in both groups (*p* ≤ 0.05).

**Figure 2:**
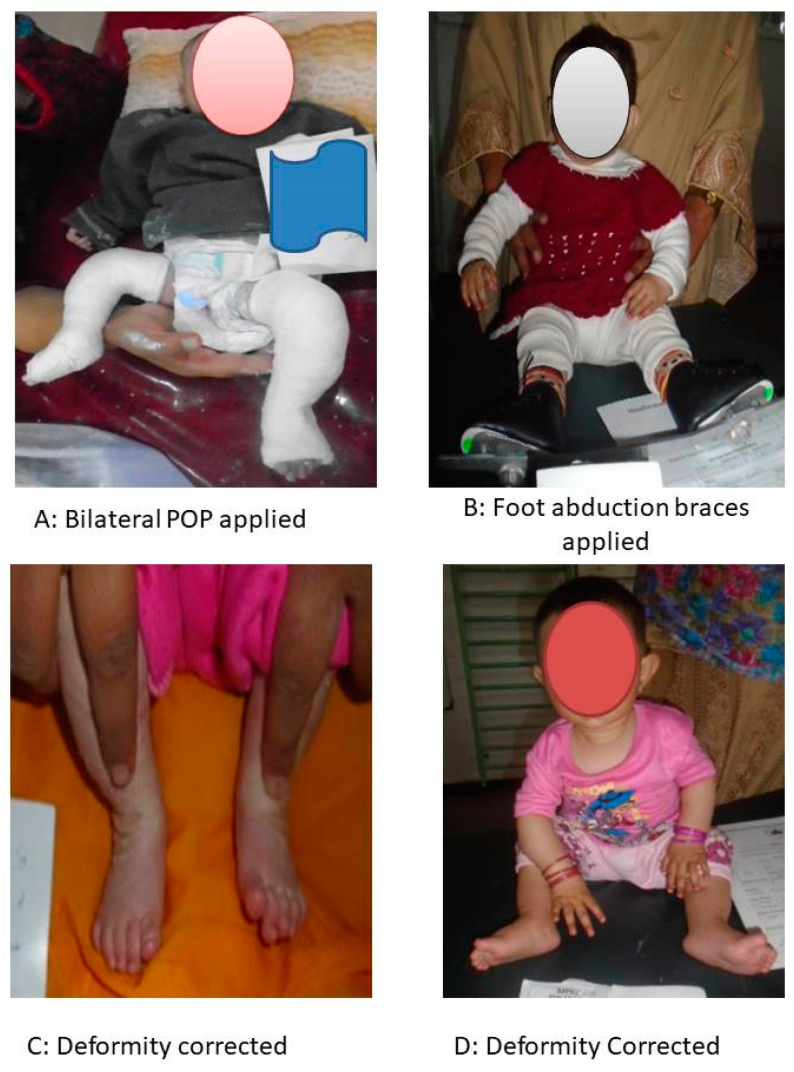
This figure shows the progress of the treatment of the child with club foot as: (**A**) after initial screening, POP was applied; (**B**) after a sufficient number of casts, the Pirani score was found to be sufficient to apply foot abduction braces. (**C**,**D**) Deformity appears to be corrected.

Progress of a baby girl with bilateral club foot from baseline to the follow up visit with its visible outcome is given as Figure 2.

**Figure 3:**
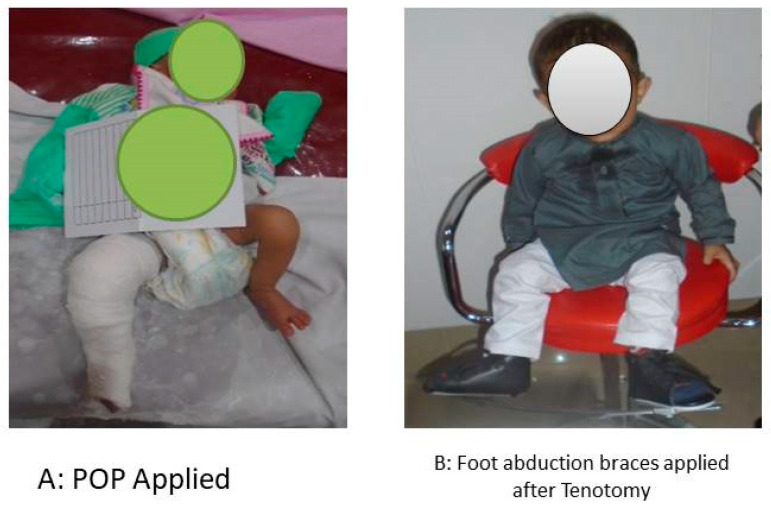
Progress of treatment. (**A**) POP was applied after initial screening. (**B**) A foot abduction brace applied after sufficient number of casts and tenotomy.

Similarly another case of a baby boy with right sided unilateral clubfoot from baseline to follow up visit is given as Figure 3.

## 4. Discussion

The Ponseti method is the gold standard for the management of children with a clubfoot deformity and can produce pain-free, normal-looking, and functional feet [1,14,17]. Our results have shown that most of the children presented with an idiopathic clubfoot deformity and the percentage of syndromic or non-idiopathic clubfoot is comparatively lower [18]. The number of casts required for syndromic or non-idiopathic clubfoot was higher than for idiopathic untreated and neglected clubfoot [19]. A tenotomy is a very important part of the corrective phase for the correction of the equinus deformity in clubfoot. The relapse of clubfoot deformity is very controversial, and there are many factors that can influence it, including age, gender, laterality, severity of the deformity, non-compliance to the foot abduction brace, baby crying, and post-surgery patient care [20,21]. This study was retrospective, and only the documented data were evaluated. It has been found that relapse can occur in both phases of the Ponseti method. In the corrective phase, relapse was reported due to a wound caused by of a broken plaster of Paris (POP) cast and poor cast hygiene. In the maintenance phase, relapse is due to poor brace compliance. Children who did not have their tenotomy conducted in the corrective phase either did not need it at the time of correction because their feet had good mobility and the Pirani score for rigid equinus was zero or they later presented with a decreased range of motion in dorsiflexion due to tight tendon achilles and toe walking. This study also found that the parents of some children who did not follow the foot abduction brace protocol experienced a complete relapse of deformity at walking age. Although certain recent studies suggest that relapse is not associated with any single factor [21]. Parents’ compliance with the foot abduction brace has been found to be associated with the outcome of the Ponseti method, but there are certain other factors like the economic status of the family of the child with clubfoot, the casting technique, cultural norms and values, the parents’ education level, and their awareness about their child’s deformity [21].

Unfortunately, we were not able to determine the factors, like casting errors, as this was a retrospective data analysis. The team working for clubfoot management was Ponseti certified and had experience of at least 7 years, so casting errors are expected to have been very unlikely. Similarly, Zhao et al. found that the relapse of clubfoot deformity is strongly associated with non-compliance with the foot abduction brace, along with other factors that include parents’ education and the annual income of the family [22]. Sangiorgio et al. also reported that poor brace compliance is the leading factor in relapse, and parents should emphasise adherence to the bracing protocol [23]. Limpaphayom et al. also reported non-compliance as a major contributing factor to the relapse of clubfoot deformity, hence supporting the results of this study [20]. Azarpira et al. concluded that relapse is associated with many factors, like the low education level of parents, prolonged follow-ups, and poor adherence to the foot abduction brace. Azarpira et al. reported that non-adherence to the foot abduction brace, prolonged follow-up time, and the mother’s education level have been associated with the relapse of clubfoot deformity [24].

This study found that poor brace compliance has been associated with crying in babies. Thus far, relatively few studies have been conducted to highlight the factors that lead to poor brace compliance. A study conducted by Memon et al. highlighted several factors that have been found to be associated with poor brace compliance. The factors included the unaffordability of time and the cost of regular follow-up [25]. None of these factors have been found to be associated with poor brace compliance in our study, as our centre provides free services to children with clubfoot except for major surgeries.

Several studies have been conducted thus far on the treatment outcomes of the Ponseti method for the management of clubfoot [8,16,17,19,26,27,28]. A study conducted by Olayinka O. Adegbehingbe et al. in Nigeria concluded that Ponseti is the best treatment option for neglected idiopathic clubfoot patients [27]. Abo El-Fadl et al. conducted a study in 2016 and concluded that the Ponseti method can improve the functional status of individuals with clubfoot and myelomeningocele [28]. M. Faizan et al. conducted research on the impacts of the Ponseti method on clubfoot in toddlers and found it very effective [26]. Jackson T. et al. proved from their research that clubfoot associated with a tethered cord requires more serial casts to achieve desirable outcomes [19]. Matar et al. conducted a research project on clubfoot associated with myelomeningocele and found that Ponseti is an effective first-line management technique, but it will require more serial casts [8,9]. Rehman et al. reported in their study that the Ponseti method is a safe, effective, and economical treatment for the treatment of idiopathic clubfoot, and it is more successful in the neonatal period [17].

A parent’s knowledge assists in getting the desired outcomes from the application of a foot abduction brace. In our centre, we received children from far-flung areas, usually villages in the mountains, where the literacy level of mothers is very low. Moreover, travelling on these uneven paths was another challenge to maintaining the position. Thus, it is suggested that parent training can help to avoid poor brace compliance. For this reason, we emphasise parents’ education regarding the deformity and importance of a foot abduction brace. Communication with parents can be very helpful for better outcomes [22,29].

Parents should be reassured about regular follow-up visits, and interactive sessions between other parents of different children with clubfoot should be arranged in order to improve brace compliance. Children with clubfoot who are at risk of relapse should be monitored closely [29].

Pavone V and co-authors reported that the Ponseti method is effective in achieving good to excellent outcomes in the performance of sports activities in children with clubfeet. There was no difference in sports performance on the basis of gender or sides involved, whether unilateral or bilateral [30].

There were many factors that were identified in this study that led to the use of foot orthoses (FO) or ankle foot orthoses (AFO) by children with clubfoot in walking age. The Ponseti protocol contraindicates ankle foot orthosis for children with clubfoot [31]. This study highlights the factors that lead to the use of foot or ankle orthoses, including different foot deformities like forefoot adduction, dynamic supination, calcaneovalgus, and hyperdorsiflexion. Children with clubfoot who faced dynamic relapse (forefoot supination or dynamic supination) were not willing to endure repetition of casts and surgeries (tibialis anterior tendon transfer). Mindler et al. reported in their study that tibialis anterior tendon transfer normalises the reoccurrence of dynamic supination in the clubfoot [32]. El-Fadl et al. also reported that tibialis anterior tendon transfer is the best method to cure the recurrence of supination in clubfoot [33]. Unfortunately, in our area, most families of children with clubfoot were not able to afford the surgery (tibialis anterior tendon transfers, re-tenotomy). For this reason, preventive daytime orthoses (AFO and FO) were made for them to prevent the deformity (dynamic supination) from getting worse. For night-time and naps, we advise keeping the foot abduction brace (Denis Brown Splint) on.

### 4.1. Limitations

The major limitation of this study is that data were collected from the file records of the patients that were maintained by the staff. It would be better to collect data directly from the patients regarding the factors related to brace compliance and relapses.

We could only use the Pirani score for measuring changes in the severity of disability in children with clubfeet; the use of other standard tools could be of value and may be considered for future prospective studies including the Clubfoot Assessment Protocol (CAP), the American Orthopaedic Foot and Ankle Score (AOFAS), and the Foot and Ankle Disability Index (FADI).

### 4.2. Recommendations

A follow-up of a longer duration until the child starts walking independently would give a wider picture of the outcomes of the Ponseti method in the management of children with clubfoot.

## 5. Conclusions

It has been concluded that the Ponseti method is effective in the management of clubfoot in terms of the Pirani score. There was a significant difference in the Pirani score between idiopathic and syndromic clubfoot and between recurrent clubfoot and syndromic clubfoot after one year of follow-up.

## Figures and Tables

**Table 1:** Baseline characteristics of the participants (*n* = 151).

	Idiopathic Untreated Clubfoot Frequency (%) *n* = 96	Recurrent Clubfoot Frequency (%) *n* = 40	Syndromic Clubfoot Frequency (%) *n* = 15
Gender distribution among different types of club foot			
Female	27 (28.1%)	6 (15.0%)	3 (20.0%)
Male	69 (71.9%)	34 (85.0%)	12 (80.0%)
Total	96 (100.0%)	40 (100.0%)	15 (100.0%)
Age (months) mean ± standard deviation			
	3.43 ± 1.14	3.35 ± 0.97	3.00 ± 0.84
	2.12 ± 2.98	6.10 ± 4.23	3.56 ± 3.56
Side(s) involved in different types of club foot			
Bilateral	52 (54.2%)	21 (52.5%)	11 (73.3%)
Right Unilateral	25 (26.0%)	13 (32.5%)	3 (20.0%)
Left Unilateral	19 (19.8%)	6 (15.0%)	1 (6.7%)
Source of referral			
Orthopedic Surgeon	42(43.8%)	3(7.5%)	7(46.7%)
pediatrician	2 (2.1%)	00 (00%)	00 (00%)
Clubfoot patient	16 (16.7%)	11 (27.5%)	1 (6.7%)
Community	36 (37.5%)	26 (65.0%)	7 (46.7%)
Total	96 (100.0%)	40 (100.0%)	15
No. of plaster casts applied to the children with club foot (mean ± std.dev)			
	7.48 ± 2.62	4.00 ± 1.63	8.46 ± 1.92

**Table 2:** Comparison of the mean ± SD of the Pirani score at baseline and after one year of follow-up between three types of clubfoot.

Idiopathic Untreated Clubfoot, Idiopathic Recurrent Clubfoot and Syndromic Clubfoot				
Time Period	Idiopathic Untreated Clubfoot	Syndromic Clubfoot	Idiopathic Recurrent Clubfoot	*p*-Value
	Mean ± SD	Mean ± SD	Mean ± SD	
Baseline	4.85 + 2.28	5.30 + 1.54	3.92 + 1.99	0.63
After one year	0.53 + 0.62	0.96 + 0.74	0.40 + 0.55	0.04 *
*p*-value	0.00	0.01	0.00	

* Significant value ≤ 0.05.

**Table 3:** Results of independent *t*-test between the Pirani score of patients treated with and without tenotomy.

*t*-Test for Equality of Means								
	Leveni’s Test for Equality of Variance						95% Confidence Interval of the Difference	
		f	Sig	t	df	*p*-Value	Lower	Upper
Total score (Rt) first visit	EVA	4.594	0.034	0.928	149	0.355	−0.42665	1.18238
	EVNA			1.048	84.942	0.298	−0.33911	1.09483
Total score (Rt) before tenotomy	EVA	8.094	0.005	5.810	149	0.000	0.22441	0.45576
	EVNA			5.312	57.325	0.000	0.21190	0.46827
Total score (Rt) before DBS	EVA	13.693	0.000	−8.497	149	0.000	−0.51314	−0.31951
	EVNA			−12.472	148.190	0.000	−0.48229	−0.35036
Total score (Rt) after one year	EVA	28.928	0.000	−3.901	149	0.000	−0.66032	−0.21628
	EVNA			−4.846	107.074	0.000	−0.61758	−0.25902
Total score (Lt) at first visit	EVA	0.057	0.812	−0.688	149	0.492	−1.32918	0.64236
	EVNA			−0.686	65.933	0.495	−1.34270	0.65589
Total score (Lt) before tenotomy	EVA	25.787	0.000	3.298	149	0.001	0.08630	0.34433
	EVNA			2.693	49.518	0.010	0.05469	0.37595
Total score (Lt) before DBS	EVA	44.558	0.000	−6.781	149	0.000	−0.43386	−0.23807
	EVNA			−9.982	148.409	0.000	−0.40248	−0.26945
Total score (Lt) after one year follow-up	EVA	2.785	0.097	−1.862	149	0.065	−1.20347	0.03566
	EVNA			−3.015	132.907	0.003	−0.96692	−0.20089

EVA: equal variances assumed; EVNA: equal variances not assumed.

## Data Availability

Due to confidentiality considerations, our data are not publicly available but can be requested from the corresponding author if necessary.
